# Electrospun Nafion^®^/Polyphenylsulfone Composite Membranes for Regenerative Hydrogen Bromine Fuel Cells

**DOI:** 10.3390/ma9030143

**Published:** 2016-02-29

**Authors:** Jun Woo Park, Ryszard Wycisk, Peter N. Pintauro, Venkata Yarlagadda, Trung Van Nguyen

**Affiliations:** 1 Department of Chemical and Biomolecular Engineering, Vanderbilt University, Nashville, TN 37235, USA; junwoo.park@vanderbilt.edu (J.W.P.); ryszard.wycisk@vanderbilt.edu (R.W.); 2 Department of Chemical and Petroleum Engineering, University of Kansas, Lawrence, KS 66045, USA; raviteja27@ku.edu (V.Y.); cptvn@ku.edu (T.V.)

**Keywords:** proton conducting membrane, electrospinning, Nafion, polyphenylsulfone, redox flow battery, regenerative fuel cell, hydrogen fuel cell, bromine

## Abstract

The regenerative H_2_/Br_2_-HBr fuel cell, utilizing an oxidant solution of Br_2_ in aqueous HBr, shows a number of benefits for grid-scale electricity storage. The membrane-electrode assembly, a key component of a fuel cell, contains a proton-conducting membrane, typically based on the perfluorosulfonic acid (PFSA) ionomer. Unfortunately, the high cost of PFSA membranes and their relatively high bromine crossover are serious drawbacks. Nanofiber composite membranes can overcome these limitations. In this work, composite membranes were prepared from electrospun dual-fiber mats containing Nafion^®^ PFSA ionomer for facile proton transport and an uncharged polymer, polyphenylsulfone (PPSU), for mechanical reinforcement, and swelling control. After electrospinning, Nafion/PPSU mats were converted into composite membranes by softening the PPSU fibers, through exposure to chloroform vapor, thus filling the voids between ionomer nanofibers. It was demonstrated that the relative membrane selectivity, referenced to Nafion^®^ 115, increased with increasing PPSU content, e.g., a selectivity of 11 at 25 vol% of Nafion fibers. H_2_-Br_2_ fuel cell power output with a 65 μm thick membrane containing 55 vol% Nafion fibers was somewhat better than that of a 150 μm Nafion^®^ 115 reference, but its cost advantage due to a four-fold decrease in PFSA content and a lower bromine species crossover make it an attractive candidate for use in H_2_/Br_2_-HBr systems.

## 1. Introduction

Renewable energy sources like wind and solar can be utilized for the generation of a significant amount of electrical energy in the United States, but their intermittent nature is hindering wide-spread implementation. The development of a suitable electrochemical energy storage system might be one solution to the above problem. Additionally, a reliable and efficient energy storage system could help in reducing electrical grid destabilization by intermittent green sources. One such system, which is scalable to the megawatt size, is a regenerative hydrogen-bromine (H_2_/Br_2_) fuel cell that utilizes Br_2_ in aqueous HBr as the oxidant. This system has several advantages over a regenerative H_2_/O_2_ fuel cell, including: (i) fast bromine oxidation/reduction kinetics which translates into low activation over-potential voltage losses, higher round trip efficiencies and a very high power density on discharge (>1.5 W/cm^2^ *versus* 0.7 W/cm^2^ for H_2_/O_2_ system) [1,2,3,4,5,6]; (ii) negligible mass transfer limitations due to the high solubility of bromine in the hydrobromic acid electrolyte; (iii) low bromine vapor pressure, which means that the bromine storage unit and the bromine electrode compartment can be operated without pressurization [7]; (iv) efficient operation with inexpensive carbon cathode, in contrast to regenerative H_2_/O_2_ fuel cells where precious metals such as Ru and Ir are required for oxygen evolution (charging) [8,9] but then Ru- and Ir-based electrodes show poor activity during discharge [8,9], which is a serious challenge for development of regenerative H_2_/O_2_ fuel cells; and finally (v) absence of carbon corrosion at the cathode during charging [10,11], which is an important advantage as carbon corrosion is a problem in regenerative H_2_/O_2_ fuel cells.

The operation of a regenerative H_2_/Br_2_ fuel cell is quite simple. During charging, hydrobromic acid (HBr) is electrolyzed to hydrogen (H_2_) and bromine (Br_2_) using electrical energy. These products are stored in external tanks until electricity is needed. During discharging, the stored products, H_2_ and Br_2_, are reacted in the fuel cell to produce HBr and electricity. The membrane-electrode assembly (MEA), a key component of the fuel cell, is composed of a polymeric proton-conducting membrane, typically selected from the perfluorosulfonic acid (PFSA) family, which physically separates the hydrogen electrode and the bromine electrode. The membrane prevents electrical shorting while providing pathways for inter-electrode proton transport and minimizing unwanted Br_2_ and Br_3_^-^ crossover. 

Nafion PFSA membranes possess good thermal/mechanical/chemical stability and high proton conductivity, and have already been utilized in hydrogen-bromine fuel cells [7,12,13,14,15]. Nafion membranes, however, suffer from high bromine species (Br^-^, Br_2_, and Br_3_^-^) crossover. The crossover causes significant columbic losses in the cell and degradation of the platinum catalyst on the hydrogen electrode [1,2,3,13]. Thus for successful deployment of efficient H_2_/Br_2_ fuel cells, a proton conducting membrane with minimal bromine species permeability is needed. 

An effective Nafion alternative should be based on a highly charged cation-exchange polymer with a high proton conductivity, which would minimize fuel cell energy losses. Unfortunately, highly charged polymers swell excessively in water and aqueous solutions and are usually brittle in the dry state. Swelling reduces the membrane’s mechanical strength and decreases the effective concentration of fixed charges thus reducing both ionic conductivity and bromine species (co-ion) exclusion. Membrane swelling can be controlled by crosslinking the polymer, but this usually exacerbates the dry membrane brittleness problem [16]. Swelling reduction can also be accomplished by blending the charged polymer with a hydrophobic/uncharged polymer, but often the two polymers are so dissimilar that the resultant phase separation negates the benefits of blending [16]. In order to improve mechanical properties and lower the swelling of highly charged polymers, Pintauro and coworkers have developed new electrospinning techniques enabling fabrication of nanofiber composite ion-exchange membranes from dissimilar polymers [17,18,19,20,21]. In particular, Ballengee and Pintauro prepared stable and mechanically robust composite proton-exchange membranes (PEMs) for hydrogen/air fuel cells using a dual-fiber electrospinning [17]. 

In the present study, a range of nanofiber composite membranes were fabricated and investigated for use in a H_2_/Br_2_ regenerative fuel cell. The membranes were composed of Nafion^®^ perfluorosulfonic acid (PFSA) ionomer for facile proton transport and uncharged polyphenylsulfone (PPSU) for mechanical reinforcement and control of membrane swelling. This paper is an extension of a previously published study on electrospun Nafion/PVDF composite fuel cell membranes [22], where PPSU is an effective reinforcement replacement for PVDF due to its excellent mechanical characteristics which enables greater control of membrane swelling.

## 2. Materials and Methods

### 2.1. Electrospinning Nafion/PPSU

Dual nanofiber mats of Nafion and PPSU were prepared by simultaneously electrospinning 1100 EW Nafion PFSA containing poly (ethylene oxide) (PEO) carrier polymer and uncharged polyphenylsulfone (PPSU), as reported previously [17]. Nafion and poly (ethylene oxide) (PEO) solutions were separately prepared by dissolving Nafion powder (prepared by evaporating the solvent from Liquion 1115, Ion Power, Inc., New Castle, DE, USA) and PEO powder (Sigma-Aldrich, St. Louis, MO, USA, 400 kDa MW) into a mixed solvent of 2:1 weight ratio n-propanol: water. These two solutions were then combined to form a Nafion/PEO electrospinning solution where PEO constituted 1 wt% of the total polymer content. For the PPSU fibers, a 25 wt% polymer solution was prepared by dissolving PPSU powder (Radel^®^ R 5500NT, from Solvay Advanced Polymers, LLC, 63 kDa MW) in a 4:1 weight ratio mixture of n-methyl-2-pyrrolidone (NMP): acetone.

**Figure 1:**
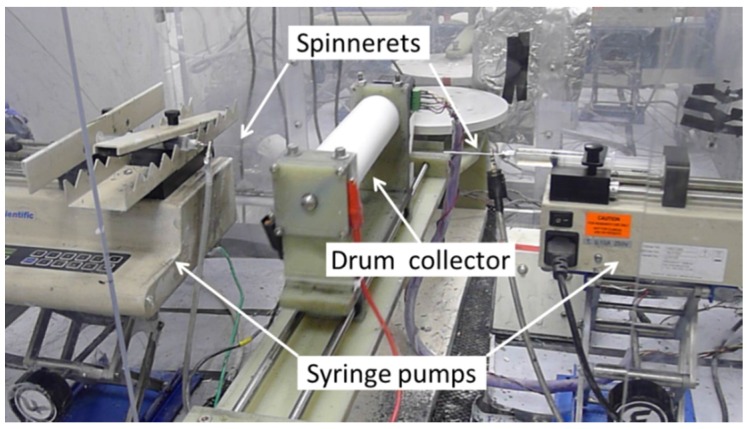
Schematic of the dual fiber electrospinning setup used in the present study.

All electrospinning experiments were carried out using a custom-built setup, shown in Figure 1, consisting of two syringes filled with the two polymer solutions and driven by two syringe pumps, two high voltage power supplies and a drum collector. The two polymer solutions were electrospun simultaneously from two separate spinnerets (stainless steel needles) placed at the opposite sides of a rotating and laterally oscillating drum collector. The Nafion/PEO solution was electrospun at the following conditions: 4.16 kV applied voltage between the needle spinneret and the drum collector surface (drum surface was grounded), 6.5 cm spinneret-to-collector distance, and a 0.2 mL/h solution flow rate. The PPSU solution was electrospun at an applied voltage of 7.5 kV, an 8.0 cm spinneret-to-collector distance, and a solution flow rate that was varied from 0.04 to 0.15 mL/h, depending on the desired mat composition. All electrospinning experiments were conducted inside a Plexiglas chamber at room temperature, with the relative humidity fixed at 35% ± 2%.

### 2.2. Dual Nanofiber Mat Processing

The Nafion/PPSU mats were processed as described earlier [17]. The dual fiber mat was first compressed four times (10 s each) at 16 kN and 25 °C. The mat was then exposed to chloroform vapor in a sealed container for 16 min which softened the PPSU and caused it to fill the voids between Nafion fibers. The membrane was then dried at 70 °C for 1 h and at 140 °C for 10 min, followed by PFSA annealing at 150 °C for 2 h under vacuum. This type of membrane will henceforth be denoted as N (fibers)/PPSU. 

Membranes with the inverse structure, which is that of Nafion reinforced by uncharged PPSU nanofibers, was also prepared, in the same manner as described by Ballengee and Pintauro [17]. The dual fiber mat was densified (compressed) at 107 kN and 127 °C for 30 s. The mat was then annealed at 150 °C in vacuum for 2 h. These membranes are denoted as N/PPSU (fibers).

Prior to testing, all membranes (nanofiber composites and a commercial Nafion^®^ 115 reference) were boiled in 1 M sulfuric acid and then in deionized water (one hour for each boiling step) to ensure full protonation of the sulfonic acid sites. The membranes were stored in deionized water at 25 °C.

### 2.3. SEM Microscopy

Electrospun mats and freeze-fractured membrane cross sections were imaged with a Hitachi S-4200 scanning electron microscope (Hitachi, Hitachinaka, Japan). The dry membrane samples were manually fractured after cooling in liquid nitrogen. The resultant micrographs were analyzed using ImageJ (version 1.38e) [23]. 

### 2.4. Ion-Exchange Capacity

Ion-exchange capacity (IEC) was determined by the standard method of acid exchange and base titration. A membrane sample of known dry weight in the acid form was soaked in 20 mL of 1 M NaCl for 3 h with stirring to exchange cations. The NaCl solution was replaced repeatedly until no H^+^ was detected in the NaCl rinsing solution. The amount of H^+^ released into the total NaCl solution volume was measured by titration with 0.01 N NaOH. The IEC of a membrane sample was calculated using Equation (1).

IEC (mequiv/g) = *VN*/*m_dry_* (1)

where *IEC* (mequiv/g) is the ion-exchange capacity (on a dry polymer weight basis), *V* (mL) is the volume of the NaOH titrating solution, *N* (mol/L) is the normality of the NaOH titrating solution, and *m_dry_* (g) is the dry mass of the membrane. The Nafion volume fraction in a composite membrane was determined from the measured IEC, as per Equation (2).

Nafion volume fraction = (*IEC_composite_*/*IEC_Nafion_*) × (*ρ_composite_*/*ρ_Nafion_*) (2)

where *IEC_composite_* and *IEC_Nafion_* are the measured ion-exchange capacity of a nanofiber composite membrane and a neat Nafion^®^ film (*IEC_Nafion_* = 0.909 mequiv/g), respectively, and *ρ_composite_* and *ρ_Nafion_* are the measured dry density of a nanofiber composite membrane and a neat Nafion^®^ film (*ρ_Nafion_* = 1.87 g/cm^3^).

### 2.5. Conductivity Measurements

In-plane ion conductivity was measured at 25 °C with rectangular pieces cut from the membranes and equilibrated with water or a 2 M HBr solution. An AC impedance method and a BekkTech, 4-electrode cell (Model—BT110, Scribner Associates, Inc. Southern Pines, NC, USA) were employed. The samples equilibrated with water were loaded into the cell and tested while fully immersed in water. Alternatively, the membranes were soaked in 2M HBr solution for 3 h and then loaded quickly into the conductivity cell, after removing excess electrolyte from the membrane surface with filter paper. Resistance was measured at a single frequency of 1 kHz and membrane conductivity was calculated using the following equation,

*σ* = *L*/(*R × w × δ*) (3)

where *σ* (S/cm) is ion conductivity, *L* (cm) is the distance between the potential sensing electrodes in the conductivity cell, *R* (Ω) is the measured resistance, *w* (cm) is the width of the membrane sample, and *δ* (cm) is its thickness. 

### 2.6. Membrane Swelling

Fully protonated composite membranes and Nafion^®^ 115 were kept in water and in 2 M HBr for at least 24 h at 25 °C prior to a measurement to ensure full equilibration. Then membrane samples were removed from the solutions and quickly wiped with a filter paper to remove surface liquid and their mass and volume were measured. Next the membranes were dried overnight at 60 °C and then for 2 h at 100 °C, and the mass and volume were re-measured. Gravimetric and volumetric swelling were calculated using Equation (4):

(4) $$\text{Membrane swelling (\%)}\;=\frac{x_{wet}-x_{dry}}{x_{dry}}\times 100$$

where *x* was either the membrane’s mass or volume. 

### 2.7. Diffusivity Measurements

**Figure 2:**
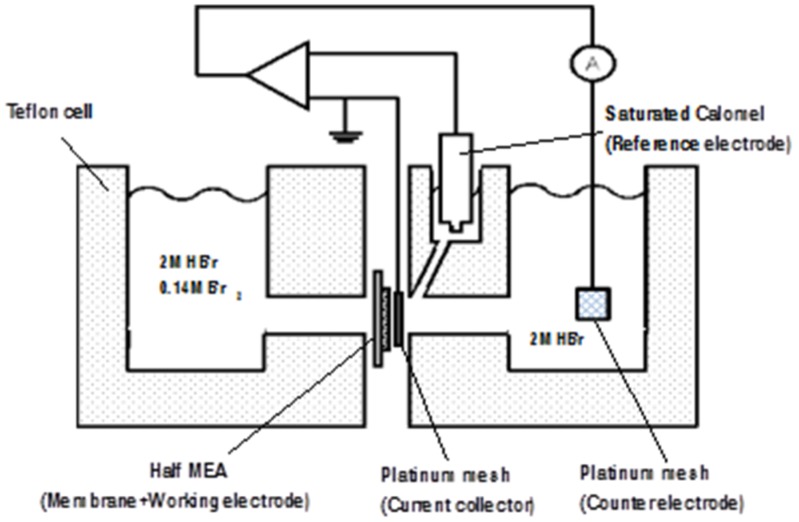
Schematic diagram of the two compartment apparatus for determining membrane diffusion coefficients.

A transient electrochemical breakthrough method [12,24,25,26] was used to determine the diffusion coefficient of Br_2_/Br_3_^-^ in membrane samples (where Br_3_^-^ is produced by the following reaction: Br_2_ + Br^-^ = Br_3_^-^). A schematic diagram of the two-compartment diffusion cell employed in the experiment is shown in Figure 2.

The downstream compartment contained a platinum mesh counter electrode and a saturated calomel reference electrode. The working Pt/C electrode was hot pressed onto the backside of a membrane to create a single electrode membrane-electrode-assembly (a half MEA). Here a standard decal method was employed where a catalyst ink electrode (0.4 mg Pt/cm^2^) was painted onto a Teflon^®^ PTFE film and then transferred to the membrane by hot-pressing at 140 °C and 0.7 MPa for 3 min. The electrode was composed of Pt/C catalyst (40% Pt/C from Johnson Matthey), 5 wt% Nafion (from a Sigma-Aldrich Nafion dispersion) and 0.2 wt% glycerol. The final Pt/C: Nafion weight ratio was 77:23.

After installing the membrane in the cell, both compartments were filled with 2 M HBr and the platinum working electrode was polarized to +0.3 V *vs.* the saturated calomel reference electrode. Once the current stabilized at 50 μA, the electrolyte in the feed compartment was rapidly drained and replaced with a pre-mixed solution of 2 M HBr with 0.14 M Br_2_.

Bromine species (Br_2_/Br_3_^-^) were electrochemically reduced as they permeated through the membrane into the downstream compartment and the resultant current transient curve was recorded using data acquisition software. All experiments were carried out at 25 °C with well-stirred solutions.

### 2.8. Analysis of Breakthrough Curves

The diffusivity of bromine species in the nanofiber composite and commercial Nafion membranes was determined by matching current *vs.* time experimental data to a theoretical transient diffusion model based on Fick’s Second Law. The differential equation and appropriate boundary and initial conditions for the diffusion cell experiment are presented below.

(5) $$\frac{\partial C}{\partial t}=D\frac{\partial^{2}C}{\partial x^{2}}$$

*C* = *C_0_* for *x* = 0 at t ≥ 0 (6)

*C* = 0 for x = *L* at *t* ≥ 0 (7)

*C* = 0 for 0 ≤ *x* ≤ *L* at *t* < 0 (8)

where *D* (cm^2^/s) is diffusivity, *C_0_* (mol/L) is the membrane-phase Br_2_/Br_3_^-^ concentration at the upstream (feed compartment) membrane/solution interface, and *L* (cm) is the membrane thickness. The downstream concentration of Br_2_/Br_3_^-^ is set equal to zero (*i.e*., all electro-reducible bromine species that diffuse through the membrane react at the downstream (sensing) Pt/C electrode that is attached to the backside of the membrane). The first term of the Laplace transform solution to Equations (5)–(8) is

(9) $$\frac{J_{t}}{J_{\infty }}=\frac{2}{\pi^{1/2}}\frac{1}{\tau^{1/2}}exp\left[-\frac{1}{4\tau }\right]$$

where *J_t_* is the current density at time t, *J_∞_* is the steady-state current density, and *τ* = *Dt/L^2^* (where KL is the membrane thickness). Also, the bromine species permeability (*P*, with units of cm^2^/s) can be calculated from the measured steady-state bromine species flux and membrane thickness,

(10) $$P=\frac{J_{\infty }L}{nFC^{b}}$$

where *n* is the number of electrons involved in the Br_2_/Br_3_^-^ reduction reaction, F is Faraday’s constant, and *C^b^* is the external (bulk) concentration of Br_2_/Br_3_^-^ (in the present study *C^b^* = 0.14 M). 

### 2.9. Fuel Cell Performance

A plain carbon paper (SGL Sigracet 10AA) was used as the Br_2_ electrode and a bi-layer gas diffusion medium consisting of carbon paper (SGL Sigracet 35BC) coated with Pt/C and Nafion binder was used as the hydrogen electrode. The Pt catalyst loading for the hydrogen electrode was approximately 0.5 mg/cm^2^. Two membrane-electrode-assemblies (MEAs) were prepared by hotpressing: one with a membrane composed of 57 vol% N (fibers)/PPSU and the other one with commercial Nafion 115 membrane. A 2 M HBr/2 M Br_2_ electrolyte mixture was fed to the Br_2_ electrode and H_2_ gas at 21 kPa was recirculated through the hydrogen electrode. The H_2_ and HBr/Br_2_ pump flow rates were 1380 cm^3^/min (97.2 A/cm^2^ equivalent) and 1.5 cm^3^/min (4.3 A/cm^2^ equivalent during discharge), respectively. In addition, liquid water at a flow rate of 0.05 cm^3^/min was injected into the H_2_ side to humidify the H_2_ gas and facilitate hydration of the Nafion ionomer binder in the hydrogen electrode. The fuel cell experiments were conducted at 25 °C and at 45 °C. 

## 3. Results and Discussion

### 3.1. Membrane Morphology

**Figure 3:**
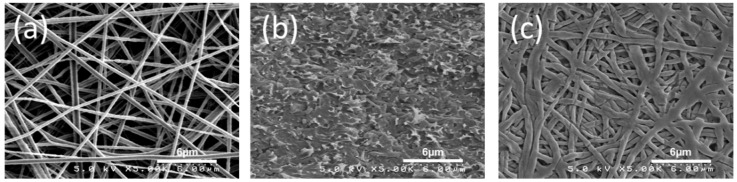
SEM images of (**a**) an electrospun Nafion/PPSU dual nanofiber mat; (**b**) freeze-fractured cross section of 57 vol% N(fibers)/PPSU; and (**c**) surface of the Nafion fiber structure after extraction of all PPSU with liquid chloroform. Magnification 5,000X.

Figure 3a shows a surface SEM image of a Nafion/PPSU dual-fiber electrospun mat, with the Nafion/PPSU ratio equal to 57/43 vol/vol and an as-spun porosity of about 80 vol%. The Nafion and PPSU nanofibers are distributed uniformly but are visually indistinguishable and the average fiber diameter is 320 nm. The dual fiber mats were processed into dense, defect free membranes via chloroform vapor exposure followed by conditioning in boiling 1 M H_2_SO_4_ and water. The cross-sectional SEM image of the fully processed membrane is shown in Figure 3b (57 vol% N(fibers)/PPSU). No evidence of defects were found in the micrographs, indicating that PPSU fibers were properly softened during the densification and solvent exposure step and formed a continuous phase within the membranes. The retention of Nafion nanofibers in the processed N (fibers)/PPSU membranes was confirmed by selectively extracting PPSU. An example of the remaining Nafion structure is shown in Figure 3c. As can be seen, a well-interconnected Nafion nanofiber network remains intact after the PPSU removal. 

### 3.2. Membrane Swelling

Gravimetric and volumetric swelling of fully protonated composite membranes and Nafion 115 were determined at 25 °C in both water and in 2 M HBr. The results are listed in Table 1. The most straightforward conclusion is that swelling of the composite membranes, whether gravimetric or volumetric, was reduced, as compared to Nafion 115. Additionally, as expected, the swelling decreased with increasing uncharged polymer (PPSU) content. For example, at 50 vol% Nafion, the electrospun composite membrane swelled 11 wt% (18 vol%) in water, which was significantly less than the swelling of Nafion 115 (28 wt% and 52 vol%). Similarly, swelling of the composite membranes in 2 M HBr was smaller than that of Nafion 115, with a nonlinear decrease in swelling with increasing PPSU content. For example, at 25 vol% Nafion, the electrospun composite membrane swelled 4 wt% (7 vol%) in 2 M HBr *versus* 20 wt% (35 vol%) swelling of Nafion 115. In summary, a significant depression in water and HBr solution uptake was observed as a result of embedding Nafion nanofibers in PPSU. This reduction in membrane swelling was important because bromine species crossover should decrease with decreasing membrane swelling. 

### 3.3. Ion Conductivity

**Figure 4:**
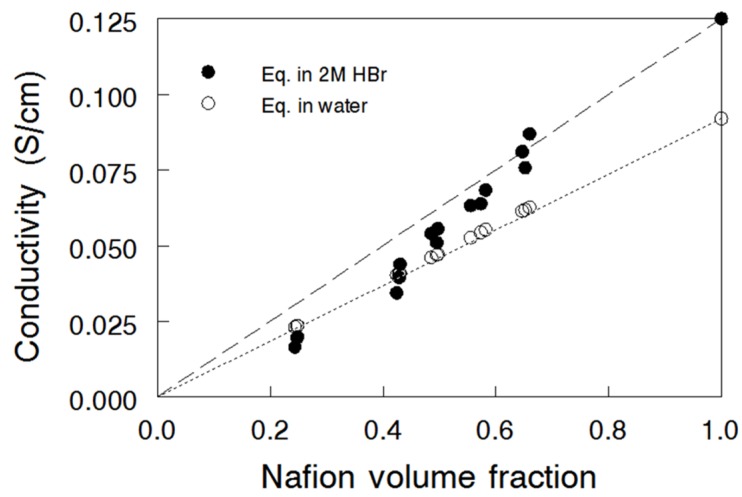
In-plane ionic conductivity of Nafion nanofiber composite membranes as a function of Nafion volume fraction at 25 °C (**○**) N(fibers)/PPSU measured in water; (●) N (fibers)/PPSU measured in 2 M HBr. The conductivity of Nafion^®^ 115 in water and in 2 M HBr is 0.084 S/cm and 0.107 S/cm, respectively.

In-plane ionic conductivity of nanofiber composite membranes equilibrated in water and in 2 M HBr was measured at 25 °C and is plotted *versus* Nafion volume fraction in Figure 4. The two points at a Nafion volume fraction of 1.0 represent the conductivity of an electrospun pure Nafion membrane, *i.e.*, and electrospun mat and processed membrane with no PPSU fibers, with conductivities of 0.092 S/cm and 0.125 S/cm in water and 2 M HBr, respectively. The high conductivity values in HBr are due to the presence of absorbed and mobile H^+^ and Br^-^ ions, *i.e.*, the increased concentration of charge carriers in the membrane leads to a higher ionic conductivity [27]. Data obtained for the reference Nafion^®^ 115 membrane are also shown for comparison; the values were slightly lower than those for the electrospun pure Nafion film (0.084 S/cm and 0.107 S/cm, for water and 2 M HBr equilibrated samples, respectively), probably due to a lower membrane swelling. A linear relationship between proton conductivity and Nafion volume fraction for the N (fibers)/PPSU membranes equilibrated in water is evident, which is in good agreement with the findings of Ballengee and Pintauro [17]. Surprisingly, the ionic conductivity of the composite membranes equilibrated in 2 M HBr did not follow a linear mixture rule (dashed line in Figure 4), especially when the Nafion volume fraction was < 0.6, where the data points lie on the conductivity curve for water equilibrated samples. This effect could be associated with the greater selectivity (Br^-^ rejection) of composite membranes with a high content of PPSU; these membranes exhibited much less swelling and thus were more effective in the Donnan exclusion of co-ions (due to an increased concentration of membrane fixed charges).

### 3.4. Bromine Species (Br_2_/Br_3_^-^) Permeation 

**Figure 5:**
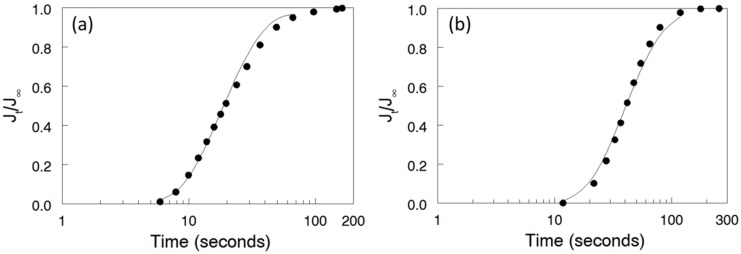
Fit of experimental data to the transient breakthrough model for: (**a**) Nafion^®^ 115; and (**b**) 57 vol% N (fibers)/PPSU nanofiber composite membrane. Figure 5a adapted with permission from ECS Transactions, 50 (2) 1217 (2012). Copyright 2012, The Electrochemical Society.

A typical fit of *J_t_*/*J_∞_* experimental data to the theoretical breakthrough curve is shown in Figure 5a for a Nafion^®^ 115 membrane [28]; only the first eight data points were fitted to maintain the accuracy of the approximation provided by the first term of the Laplace transform solution to the Fick’s equation (Equation (9)) [24]. Based on the fitted curve, the Br_2_/Br_3_^-^ diffusion coefficient was calculated equal to 1.45 × 10^−6^ cm^2^/s, as reported earlier [28]. Similar transient curve experiments were performed with selected electrospun membranes. As an example, the fit of experimental *vs.* time data to the theoretically predicted breakthrough curve is shown in Figure 5b for the 57 vol% N (fibers)/PPSU membrane. The resultant diffusion coefficient of bromine species (Br_2_/Br_3_^-^) in the composite membrane was 7.79 × 10^−8^ cm^2^/s, significantly lower than the value of 1.45 × 10^-6^ cm^2^/s for Nafion^®^ 115.

**Figure 6:**
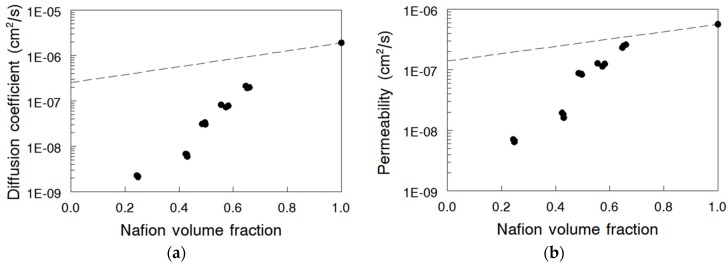
(**a**) Diffusion coefficient of bromine species (Br_2_ and Br_3_^-^) in Nafion nanofiber composite membranes of different Nafion volume fractions at 25 °C. Diffusion coefficient of Nafion^®^ 115 was 1.45 × 10^−6^ cm^2^/s; (**b**) Steady-state permeability of bromine species (Br_2_ and Br_3_^-^) for the nanofiber composite membranes as a function of Nafion volume fractions at 25 °C. Steady-state permeability of Nafion^®^ 115 was 4.26 × 10^−6^ cm^2^/s.

Br_2_/Br_3_^-^ diffusion coefficients of all nanofiber composite membranes are plotted in Figure 6a as a function of Nafion volume fraction. An increase in membrane PPSU content led to a significant reduction in the Br_2_/Br_3_^-^ diffusion coefficient, from 1.90 × 10^−6^ cm^2^/s for pure electrospun Nafion to 2.20 × 10^−9^ cm^2^/s for the membrane with 75 vol% PPSU. Comparing the above data with the diffusion coefficient obtained for N (fibers)/PVDF membranes in [18], it can be concluded that replacing the PVDF matrix with PPSU led to a 35-fold reduction in Br_2_/Br_3_^-^ diffusion coefficient at 25 vol% Nafion content. Similarly, there was 15-fold diffusion coefficient reduction when PVDF was replaced by PPSU at 50 vol% Nafion. These differences were attributed to the higher swelling of the N (fibers)/PVDF membranes in 2 M HBr, as compared to the swelling of N (fibers)/PPSU composites at the same Nafion content.

The experimentally determined steady-state Br_2_/Br_3_^-^ membrane permeability is plotted as a function of Nafion volume fraction in Figure 6b. As expected, the permeability decreased with increasing content of the uncharged polymer following the same trend as the Br_2_/Br_3_^-^ diffusion coefficient. The decrease is most likely related to an increase in tortuosity and decrease of the cross-sectional area for diffusion, along with the concurrent swelling reduction, all of which contributed to improvement in membrane’s Br_2_/Br_3_^-^ barrier properties. It was noted that both the diffusion coefficient and permeability were lower for Nafion 115 membrane compared to those values for membrane from electrospun and densified Nafion (without PPSU). This could indicate either a reduced level of crystallinity or the presence of some residual porosity in the processed electrospun films, where the latter could have occurred during extraction of the PEO carrier polymer. 

The concept of relative selectivity, as proposed by Cussler and coworkers for characterization of direct methanol fuel cell membranes [29], was utilized to characterize the nanofiber composite membranes. Taking Nafion^®^ 115 as the reference, the relative selectivity is defined by Equation (11) [30].

(11) $$\text{Relative selectivity}=\frac{{\left[\frac{\kappa }{P}\right]}_{composite\;membrane}}{{\left[\frac{\kappa }{P}\right]}_{Nafion\;115}}$$

**Figure 7:**
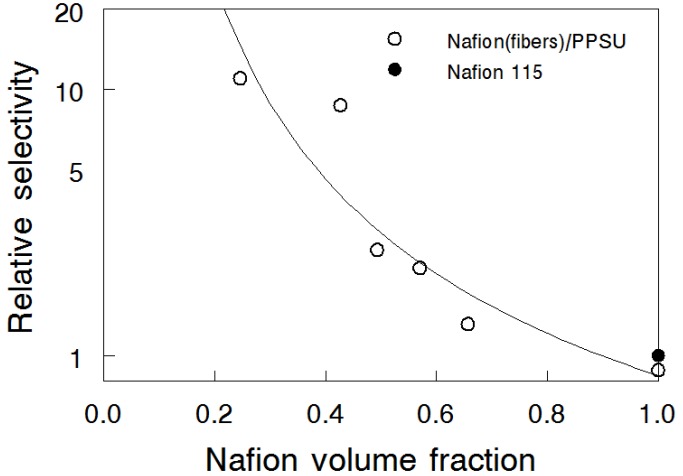
Relative, with respect to Nafion 115, selectivity of the nanofiber composite membranes as a function of Nafion volume fractions at 25 °C. (○) N(fibers)/PPSU; (●) Nafion^®^ 115.

The ionic conductivities (*κ*, S/cm) from Figure 4 and the permeabilities (*P*, cm^2^/s) from Figure 6b were combined to calculate relative membrane selectivity, which is plotted as a function of Nafion volume fraction in Figure 7. It can be seen that as the Nafion content decreased, the membrane selectivity increased nonlinearly, because the drop in bromine species (Br_2_/Br_3_^-^) crossover was greater than the decrease in the ionic conductivity. For example, the 25 vol% N (fibers)/PPSU membrane had selectivity of 11.0, which means that bromine species crossover flux would be that much lower compared to Nafion 115, if the thickness of the composite membrane were adjusted so that its area specific resistance matched that of Nafion 115. A 25 vol% N (fibers)/PPSU membrane with a thickness of 2.5 μm had a crossover flux equal to that of Nafion 115 but the area specific resistance (ASR) of the membrane was about 10-times smaller. Similarly, a 25 vol% N (fibers)/PPSU membrane with a thickness of 25 μm would have ASR equal to that of Nafion 115 but the crossover flux of the membrane would be an order of magnitude smaller. 

### 3.5. Nafion Nanofiber Composite Membrane Stability in 2 M HBr-0.14 M Br_2_

**Figure 8:**
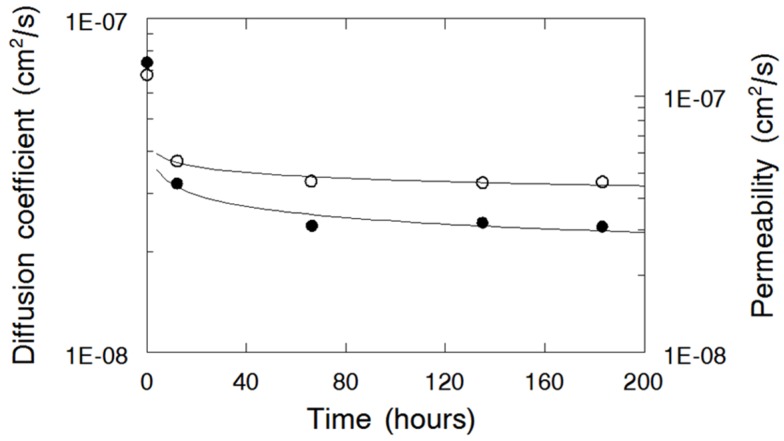
Diffusion coefficient (●); and steady-state permeability (○) of Br_2_/Br_3_^-^ in N(fibers)/PPSU nanofiber composite membrane as a function of soak time in a solution of 0.14 M Br_2_ in 2 M HBr at 25 °C.

Membrane chemical stability was evaluated by measuring ion conductivity, diffusion coefficient, and steady-state permeability of membranes immersed in 0.14 M Br_2_ in 2 M HBr at 25 °C for 12, 66, 135, and 183 hours. These tests were performed with a 55 vol% N (fibers)/PPSU membrane. It was found that the ionic conductivity remained constant at 0.058 S/cm during this soaking test, which indicated that –SO_3_H ion exchange groups bonded to the carbon-fluorine side chains of Nafion were stable in a bromine environment [31]. As seen in Figure 8, the diffusion coefficient and the steady-state permeability for the membrane decreased after 12 h and then stabilized at 2.40 × 10^−8^ cm^2^/s and 4.60 × 10^−8^ cm^2^/s respectively, which might indicate some kind of short-term structural rearrangement (densification) and/or chemical reaction between bromine and PPSU. In addition, no irreversible color change of the membranes was observed.

### 3.6. Bromine Species (Br_2_/Br_3_^-^) Permeation for the Two Complementary Nanofiber Composite Membrane Structures

**Figure 9:**
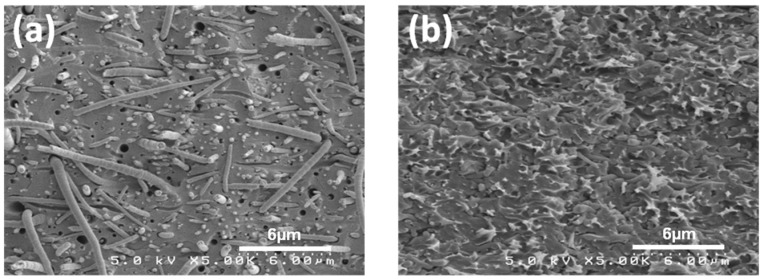
SEM micrographs of the cross-sections of two 57 vol% Nafion/PPSU membranes with complementary morphologies: (**a**) N/PPSU(fibers)–PPSU nanofibers embedded in a Nafion matrix; and (**b**) N(fibers)/PPSU–Nafion nanofibers embedded in a PPSU matrix. Magnification is 5,000X.

Figure 9 shows freeze-fractured SEM cross section images of N/PPSU (fibers) (Figure 9a) and N (fibers)/PPSU (Figure 9b) composite membranes, where both membranes were of the same composition (57 vol% Nafion and 43 vol% PPSU). The presence of nanofibers, either PPSU (Figure 9a) or Nafion (Figure 9b), embedded within a continuous matrix of the second membrane component (Nafion in Figure 9a, or PPSU in Figure 9b) is evident. Ionic conductivity and transport properties for both the “normal” structure (a reinforcing PPSU matrix and Nafion fibers) and the complementary/inverse structure (a membrane with reinforcing PPSU fibers and a Nafion matrix) are summarized in Table 2. As expected based on earlier studies [17,22], the composite membranes with PPSU nanofibers exhibited a higher diffusion coefficient and steady-state permeability, as compared to those measured for the membrane with Nafion nanofibers of the same composition. A similar finding was obtained for hydrogen/bromine fuel cell membranes composited of N (fibers)/PVDF and N/PVDF (fibers) [22]. The difference in Br_2_/Br_3_^-^ permeability between the two membrane morphologies was associated with differences in swelling, which did not change the proton transport rate but affected the diffusivity of the much bulkier bromine species.

### 3.7. H_2_-Br_2_ Regenerative Fuel Cell Performance 

**Figure 10:**
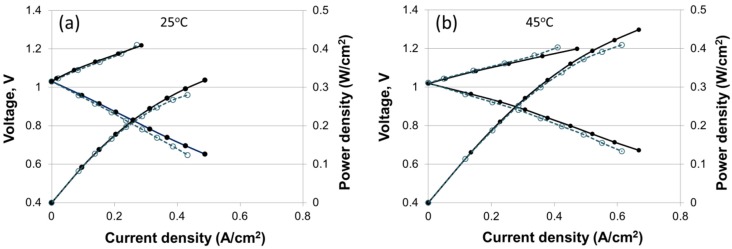
H_2_/Br_2_ regenerative fuel cell performance at 25 °C (**a**) and 45 °C (**b**) with a 55 vol% N (fibers)/PPSU nanofiber composite membrane, 65 μm in thickness (●); and Nafion 115 membrane, 140 μm in thickness (○). Plots show both charging and discharging curves [34]. Adapted with permission from *J. Electrochem. Soc.*, 162, F919 (2015). Copyright 2015, The Electrochemical Society.

The H_2_-Br_2_ regenerative fuel cell experiments (charging and discharging) were performed at 25 °C and 45 °C with an electrospun dual fiber 55 vol% N (fibers)/PPSU nanofiber composite membrane and with Nafion 115, which served as a reference. The resultant current–voltage curves are shown in Figure 10. The first thing that can be noticed is the absence, in the discharge curves, of a vertical drop in the cell voltage at low current density as observed in a typical H_2_/O_2_ fuel cell. This is the result of fast kinetics at both the negative (H_2_) and the positive (Br_2_) electrode. The kinetic losses contribute only a small fraction of the overall voltage loss in a H_2_/Br_2_ fuel cell, which is in contrast to the significant losses at the O_2_ electrode due to the sluggish ORR kinetics. Therefore, in H_2_/Br_2_ fuel cell, the performance is limited mainly by the ohmic resistance in the system (predominantly membrane) and bromine species crossover [32].

The electrospun membrane thickness was 65 μm and its area-specific resistance (ASR) was equal to that of Nafion 115. As shown in Figure 10, the performance of electrospun membrane was somewhat better compared to that of Nafion 115 at both 25 °C and 45 °C. At 25 °C the maximum power densities were 0.32 W/cm^2^ and 0.28 W/cm^2^ for the composite and Nafion membrane, respectively. At 45 °C, the maximum power densities were 0.45 W/cm^2^ and 0.41 W/cm^2^ for the composite and Nafion membrane, respectively. The diffusivity and steady-state permeability of bromine species in the composite membrane were lower than those of commercial Nafion 115, but there was no significant difference in the open circuit voltage (OCV) for the two membranes. 

Utilization of thinner membranes would lead to even better power output however the price would be an increase in bromine species (Br_2_, Br^-^, and Br_3_^-^) crossover, resulting in poisoning and corrosion of hydrogen electrode catalyst (Pt), particularly during H_2_ supply interruption at cell startup/shutdown and during charging [33]. Thus, the fuel cell lifetime would be reduced if a too thin membrane were used. A more extensive discussion/analysis of H_2_-Br_2_ fuel cell performance with dual-fiber N (fibers)/PPSU membranes can be found in [34].

## 4. Conclusions

Nanofiber composite membranes were fabricated from electrospun Nafion/polyphenylsulfone (PPSU) dual-fiber mats for use in a regenerative hydrogen bromine (H_2_/Br_2_) fuel cell. The resultant structures consisted of Nafion nanofibers surrounded by uncharged PPSU and PPSU nanofibers surrounded by Nafion. All membranes were stable in 0.14 M Br_2_-2M HBr aqueous solutions. Based on experimentally measured ionic conductivities and bromine species permeabilities, relative selectivities were calculated (where the selectivity is the ratio of conductivity to permeability, as compared to the same ratio of a Nafion 115 reference). Five important conclusions can be made based on the experimental results: (1) composite nanofiber membranes had better selectivities than Nafion 115, e.g., 2.5 and 11.0 for the membranes containing 50 vol% and 25 vol% Nafion fibers embedded in PPSU matrix, respectively; (2) when the PPSU content of a composite membrane was increased, there was a decrease in ionic conductivity, but an even greater reduction in bromine species permeability, so the relative selectivity increased with increasing uncharged PPSU polymer content; (3) composite membranes with Nafion nanofibers embedded in PPSU matrix had a lower bromine species permeability, as compared to membranes of similar Nafion content where PPSU nanofibers were embedded in a Nafion matrix; (4) composite membranes with Nafion nanofibers and PPSU matrix had a lower bromine species permeability as compared to previously reported nanofiber composite membranes where Nafion fibers were embedded in a PVDF matrix (where both the PPSU and PVDF based films had the same ionic conductivity); and (5) H_2_/Br_2_ charge/discharge regenerative fuel cell experiments at 25 °C and 45 °C showed that the power output with 65 μm thick nanofiber membrane containing 55 vol% Nafion fibers was ~10% higher than that with a 150 μm thick Nafion^®^ 115 reference. Taking into account the lower bromine species crossover of nanofiber composite membranes and significant cost advantage of such films, due to their relatively low PFSA content, it can be concluded that electrospun composite Nafion/PPSU membranes are attractive candidates for use in H_2_/Br_2_ grid-scale regenerative fuel cell storage systems. 

## Figures and Tables

**Table 1:** Swelling in water and in 2 M HBr at 25 °C of Nafion/PPSU composite membranes and Nafion^®^ 115.

Membrane	Mass Swelling (%)		Volume Swelling (%)	
	Water	2 M HBr	Water	2 M HBr
Nafion^®^ 115	28	20	52	35
N (fibers)/PPSU	–	–	–	–
57 vol% Nafion	14	13	20	15
50 vol% Nafion	11	12	18	13
44 vol% Nafion	8	8	11	12
25 vol% Nafion	4	4	7	7

**Table 2:** Transport properties of the two nanofiber composite membrane structures, with the same Nafion content. The measured properties of a Nafion 115 reference film are also listed.

Structure		Ion Conductivity (mS/cm)	Diffusion Coefficient (cm^2^/s)	Permeability (cm^2^/s)	Relative Selectivity
N/PPSU		–	–	–	–
57 vol% Nafion	Nafion fibers	65	7.36 × 10^−8^	1.22 × 10^−7^	2.1
	PPSU fibers	65	1.41 × 10^−7^	2.11 × 10^−7^	1.2
Nafion 115		107	1.45 × 10^−^^6^	4.26 × 10^−7^	1.0

## Abbreviations

The following abbreviations are used in this manuscript:

ASR: Area specific resistance
MEA: Membrane electrode assembly
N: Nafion®
OCV: Open circuit voltage
PEO: Polyethylene oxide
PFSA: Perfluorosulfonic acid
PPSU: Polyphenylsulfone
PVDF: Polyvinylidene fluoride
SEM: Scanning electron microscopy
